# Risk of long-term infection-related death in clinical osteoporotic vertebral fractures: A hospital-based analysis

**DOI:** 10.1371/journal.pone.0182614

**Published:** 2017-08-09

**Authors:** Ying-Chou Chen, Wei-Che Lin

**Affiliations:** 1 Department of Rheumatology, Kaohsiung Chang Gung Memorial Hospital, Chang Gung University College of Medicine, Kaohsiung, Taiwan; 2 Department of Radiology, Kaohsiung Chang Gung Memorial Hospital, Chang Gung University College of Medicine, Kaohsiung, Taiwan; Seoul National University College of Medicine, REPUBLIC OF KOREA

## Abstract

**Background:**

Osteoporotic vertebral fractures adversely impact quality of life and also increase the risk of infection and mortality. Alendronate treatment increases bone mass and reduces the risk of fractures in patients with osteoporosis by suppressing bone resorption. We investigated the relationship between alendronate treatment and infection-related death in patients with osteoporotic vertebral fractures.

**Methods:**

We retrospectively reviewed patients with osteoporosis and vertebral fractures from January 2001 to December 2007. The use of alendronate, glucocorticoid and medical factors including smoking, alcohol consumption, diabetes, hypertension, stroke, liver disease, heart disease, and pulmonary disease were analyzed. Cox regression was used to analyze the factors associated with life-threatening infections.

**Results:**

A total of 210 patients (161 females and 49 males) were included with a mean age of 74.06±7.43 years. Among them, 87 had life-threatening infections and 123 did not. In Cox regression analysis, the patients who used alendronate had a significantly lower risk of life-threatening infections (*p* = 0.006, HR = 0.845, 95% CI 0.750–0.954), while glucocorticoid users had higher risk of death (*p* = 0.010, HR = 2.037, 95% CI 1.187–3.498).

**Conclusions:**

Osteoporosis was associated with a high rate of life-threatening infections, and the use of alendronate had a lower rate of infection-related death. Therefore, we suggest that alendronate be used after vertebral fractures in these patients.

## Introduction

Osteoporotic fractures of the spine are a problem related to aging, and 18% of women and 11% of men will develop symptomatic vertebral compression fracture [1]. It is reported that vertebral fractures increase overall mortality up to 15% [2]. The most common long-term cause of death in patients with vertebral fractures is infection-related mortality [3]. Because severe injury of a vertebral body may result in a prominent increase in interstitial space, failure to achieve union and pseudo-arthritis can occur at the site of the fractured endplate [4]. Likewise, severe bone marrow edema and a greater propensity to develop spine refractures may arise. Therefore, an increase in the incidence of fractures causes more patients to be bed ridden and hospitalized and limits chest expansion [5], which leads to an increase risk of infection.

Alendronate treatment increases bone mass and reduces the risk of fractures in patients with osteoporosis by suppressing bone resorption. Therefore, the aim of this study was to investigate the relationship between alendronate treatment and infection-related deaths in patients with osteoporotic vertebral fractures and the associated risk factors.

## Materials and methods

The study design was approved by the institutional review board at Kaohsiung Chang Gang Memorial Hospital. It was a retrospective review of patients with osteoporosis with acute non-traumatic vertebral fractures. Inclusion criteria: 1) Osteoporosis with acute vertebral fractures; 2) Magnetic resonance imaging (MRI) showed low signal intensity (SI) on T1-weighted images and enhanced SI on T2-weighted images, and enhanced fat-suppressed SI on T1-weighted images of the injured vertebral body at the injured vertebral body [6]; and 3) Those undergoing vertebroplasty. Exclusion criteria: 1) Those with recent systemic infection in one week; and 2) Malignant neoplasia. The definition of infection-related death was after long-term follow up, and the cause of death was infection-related such as pneumonia or urinary tract infection.

All of the patients included in the study underwent baseline bone density studies, and age, gender, and body mass index (kg/m^2^) were recorded. All associated medical diseases such as diabetes, hypertension, and liver and renal diseases and anti-osteoporotic medication (alendronate) were recorded. The period of follow-up for each participant was calculated as the time from inclusion in the study to the time of death or the end of the study (December 2014), whichever occurred first.

### Statistical analysis

Statistical analysis was performed using SPSS software, version 22.0 (SPSS, Chicago, IL, USA). Patient characteristics were reported as simple descriptive statistics (i.e., mean ± standard deviation [SD]). Comparisons between independent means were analyzed using the independent t-test. Relationships between categorical variables were evaluated using the chi-square test. Cox regression analysis was used to adjust for potential confounding factors. Statistical significance was set at *p*<0.05.

## Results

In total, 210 patients with MRI-proven acute vertebral fractures who underwent vertebroplasty were included. Of these, 87 had infection-related deaths including 25 instances of pneumonia, 3 urinary tract infections, 1 acute cholecystitis and 58 cases of unknown primary septic shock. The mean follow-up period was 6.71±3.83 years. All of the patients had grade 3 vertebral fractures using a semiquantitative grading scale, and a T score < -2.5 according to bone densitometry. There were no significant differences in age, body mass index, gender, number of vertebral fracture, and underlying medical illnesses between the patients with and without life-threatening infections (Table 1). Glucocorticoid users had a higher rate of infection-related death, while the rate for alendronate users was lower.

**Table 1:** Characteristics of the patients who did and did not have life-threatening infections.

Variables	Infection-related deaths (n = 87)	No infection-related deaths (n = 123)	*P-*value
Age (years)	74.71±8.02	73.61±6.98	0.291
Body mass index (kg/m^2^)	22.63±5.26	23.39±4.48	0.256
Gender (Female %)	66(75.9)	95(77.2)	0.817
Spine fracture (number)	1.89±0.98	1.86±1.26	0.82
Smoking	12(13.8)	10(8.1)	0.253
Alcohol consumption	6(6.9)	4(3.3)	0.325
Rheumatoid arthritis	5(5.7)	6(4.9)	0.765
Diabetes mellitus (%)	29(33.3)	29(23.6)	0.158
Hypertension	50(57.5)	60(48.8)	0.262
Cardiovascular disease	1(1.1)	7(5.7)	0.144
Pulmonary disease	7(8.0)	7(5.7)	0.579
Liver disease	4(4.6)	5(4.1)	0.555
Glucocorticoid use	22(25.3)	15(12.2)	0.017
Alendronate use	25(28.7)	59(48.0)	0.006

When we adjusted for potential confounding factors such as smoking, alcohol consumption, diabetes, hypertension, cardiovascular disease, pulmonary disease, liver disease, and kidney disease, the treated patients still had a lower mortality rate than did those who did not receive treatment (*p* = 0.006, HR = 0.845, 95% CI 0.750–0.954). In comparison, those who received glucocorticoid therapy had a higher rate of infection-related death than those who did not receive glucocorticoid therapy (*p* = 0.010, HR = 2.037, 95% CI 1.187–3.498) (Table 2). When we compare crude HR and adjusted HR, alendronate use still had a lower risk to decrease risk of life-threatening infection at crude HR and adjusted HR (Table 3).

**Table 2:** Multivariate analysis of the hazard ratios for life threatening infections.

	p value	HR (95% CI)
Age	0.148	1.024(0.992–1.057)
Body mass index (kg/m2)	0.464	0.982(0.937–1.030)
Gender	0.437	0.776(0.410–1.470)
Smoking	0.641	1.245(0.495–3.131)
Alcohol consumption	0.212	2.122(0.652–6.915)
Rheumatoid arthritis	0.664	0.786(0.265–2.329)
Diabetes	0.067	1.570(0.969–2.544)
Hypertension	0.629	1.119(0.709–1.766)
Cardiovascular disease	0.088	0.176(0.024–1.290)
Pulmonary disease	0.507	1.312(0.589–2.925)
Liver disease	0.264	1.863(0.626–5.548)
Glucocorticoid use	0.01	2.037(1.187–3.498)
Alendronate use	0.006	0.845(0.750–0.954)

**Table 3:** Comparison of crude HR and adjusted HR. Alendronate use leads to stably decrease risk of life-threatening infection at crude HR and adjusted HR.

	Crude HR (95% CI)	Adjusted HR (95% CI)
Age	1.011(0.983–1.039)	1.024(0.992–1.057)
Body mass index (kg/m2)	0.983(0.941–1.026)	0.982(0.937–1.030)
Gender	1.073(0.676–1.701)	0.776(0.410–1.470)
Smoking	1.615(0.916–2.846)	1.245(0.495–3.131)
Alcohol consumption	1.443(0.631–3.297)	2.122(0.652–6.915)
Rheumatoid arthritis	0.923(0.375–2.272)	0.786(0.265–2.329)
Diabetes	1.496(0.982–2.278)	1.570(0.969–2.544)
Hypertension	1.191(0.798–1.778)	1.119(0.709–1.766)
Cardiovascular disease	0.195(0.027–1.402)	0.176(0.024–1.290)
Pulmonary disease	1.077(0.499–2.325)	1.312(0.589–2.925)
Liver disease	2.085(0.911–4.773)	1.863(0.626–5.548)
Glucocorticoid use	1.824(1.149–2.896)	2.037(1.187–3.498)
Alendronate use	0.423(0.270–0.664)	0.845(0.750–0.954)

HR: Hazard ratio.

## Discussion

Increased mortality has been found in osteoporotic patients [7,8]. Further, osteoporotic related vertebral deformities have been reported to increase mortality and fracture [9–14]. Respiratory restriction may contribute to death in patients with severe vertebral fracture [15].

In this study, the patients who used alendronate had a significantly lower risk of life-threatening infections. In our previous report, those using alendronate can significantly reduce adjacent fracture after vertebroplasty (P = 0.011) [16].

Anti-osteoporotic therapy has been reported to reduce mortality in patients with osteoporosis who are at a high risk of fractures [17–19]. Several agents have been used for the treatment of osteoporosis, including: bisphosphonates (alendronate, ibandronate, risedronate, and zoledronic acid), calcitonin, selective estrogen receptor modulators (raloxifene), parathyroid hormone (teriparatide), and RANK-ligand inhibitors (denosumab) [20]. However, bisphosphonates are most commonly used to treat osteoporosis.

In this study, the patients who received alendronate therapy had a 15.5% reduction in infection-related death [adjusted HR 0.845(95% CI 0.750–0.954)]. To the best of our knowledge, this is the first long-term study specifically designed to examine the association between alendronate therapy and the risk of life-threatening infections in patients with vertebral fractures.

The possible mechanisms of alendronate therapy to reduce the risk of infection-related death are multifactorial. One possible reason may be by preventing new fractures [21]. A study on zoledronic acid revealed that the reduction in the risk of fractures can decrease mortality by 28% [22]. Second, bisphosphonate had effects not only on bone resorption, but also on extraskeletal effects [23,24]. Bisphosphonate can influence the production of pro- and anti-inflammatory cytokines (γδ T cells, TNF-α, and interferon-γ). In a zoledronic acid study, pneumonia-related deaths were reduced, which further supports this hypothesis [22]. Third, calcified blood vessels take up bisphosphonates and their high affinity for mineralized tissue, and the impact on local nitric oxide generation may inhibit the mevalonate pathway in vessel walls [25]. This will influence the atherogenic process, monocyte adhesion to the endothelial surface, vascular smooth muscle cell proliferation, platelet aggregation, and vasoconstriction [26]. So, a statin-like effect of bisphosphonates might explain some of their effects on infection-related mortality.

Glucocorticoid could impair cellular and humoral immune functions and it was found that prednisone doses over 7.5–10 mg/day are well-recognized risk factors for infection [27]. So, it is important to stop administering glucocorticoid as soon as possible to decrease the risk of infection.

There are several limitations to this study. First, as an observational study, treatment was not randomly allocated. The group that received treatment was possibly a self-selected and health-oriented one. Second, the sample size was small, therefore many risk factors (i.e., age and diabetes) were not found to be significant. Third, because the study had a retrospective design, we could not include data such as the use of vitamin D and calcium supplements. Fourth, immortal time bias could not be excluded. But those with alendronate exposure may prolong after 1 month of vertebroplasty therapy, so in these instances, it was untreated and will interfere the results of the judgment. In our series, however, most of the treated patients received alendronate immediately after vertebropalsty therapy, so the confounding factor of the immortal bias was very small. Fifth, while recall bias may be found in a retrospective study, we used standardized data collection protocols: information about exposure should be collected in the same way and at similar timing for alendronate use or not. Sixth, a potential problem of hospital-based studies is the selection bias. As a result, we attempted to collect as large a sample as possible to minimize the selection bias. Seventh, only grade 3 vertebral fractures were used. However, this was the local policy, because grade 1 and 2 vertebral fractures did not need to accept vertebroplasty. Finally, most of the patients were female which may limit generalizations to males, but vertebral fractures were predominant in females in the general population [1], so the limitation here is very low. This study also has a number of strengths. For instance, baseline MRI scans were taken for each participant, all of whom had clinically diagnosed vertebral fractures. Thus, we were able to exclude other secondary causes of vertebral fractures such as cancer or pyogenic infections.

## Conclusions

In summary, our results suggest that alendronate therapy is associated with lower risk of infection-related death after vertebral fracture, while glucocorticoid increases the risk of infection-related death. The beneficial effect of alendronate remained even after adjusting for a large number of factors. So, we recommended stopping glucocorticoid and adding alendronate to decrease the risk of infection-related death.
